# The Use of Microwave Treatment as a Sustainable Technology for the Drying of Metallurgical Sludge

**DOI:** 10.3390/ma17246207

**Published:** 2024-12-19

**Authors:** Marta Ślęzak, Piotr Migas, Mikolaj Bernasowski

**Affiliations:** Faculty of Metals Engineering and Industrial Computer Science, AGH University of Krakow, Mickiewicza 30, 30-059 Krakow, Poland; mslezak@agh.edu.pl (M.Ś.); pmigas@agh.edu.pl (P.M.)

**Keywords:** lagoon sludge, iron-bearing waste, microwaves

## Abstract

The modern metallurgical industry produces approximately 90% of the volume of all produced steel; for this, integrated technology based on fossil materials such as coal, fluxes, and especially iron ore is used. This industry generates large amounts of waste and by-products at almost all stages of production. Alternative iron and steel production technologies based on iron ore, methane, or pure hydrogen are also not waste-free. To ensure sustainable waste management, efforts are made to seal processes as well as capture and recycle dusty waste. This work presents the results of research on the processing of sludge resulting from the dedusting of the basic oxygen furnace (BOF) process and landfilling in a lagoon. The work discusses the treatment of fine dusty sludge hydrated to 26–60% H_2_O, to which various amounts of caking agents were added; also discussed are the rheological characteristics of the tested suspension systems, the possibility of forming these systems into larger fractions, and rapid drying using 100–600 W microwaves with a drying time of 1–9 min. The aim was to identify, describe, and characterize the parameters of the agglomeration process and obtain a product that was durable enough to transport and dose into slag baths in order to reduce iron oxides in liquid phases. During the research, completely dried briquettes with an appropriate strength were obtained. The study demonstrates that microwave drying at 300 W for 6 min achieved complete drying with a weight loss of 35%, whereas a higher-power treatment at 750 W for 2 min enhanced compressive strength by up to 95% and reached 15 N/psc, which was comparable with green iron ore pellets. This approach offers a sustainable alternative to traditional methods, but with a reduced drying time.

## 1. Introduction

The steel industry is important for sustainable growth, added value, and quality employment in the economy. Steel is a material that enables the implementation of green energy technology; it is infinitely recyclable and its residues and waste can become valuable resources, thus contributing to the creation of a circular economy [1,2,3].

In 2019, 158.8 million tons of crude steel were produced in Europe, including 93.9 million tons (59.1%) in blast furnaces (BFs) and basic oxygen furnaces (BOFs) and 64.9 million tonnes (40.9%) in electric arc furnaces (EAFs) from scrap metal. A total of 426 kg of waste per tonne of liquid steel (average values) is generated by the BF–BOF route, while 185 kg of waste is generated per tonne of liquid steel from the EAF process [1].

Taking these details into account, the amount of by-products created in 2019 was approximately 52 million tonnes, including ~40 million tonnes from BF–BOF and ~12 million tonnes via EAFs. The largest proportion of these by-products comprised slag fractions (mainly from BFs, BOFs, and EAFs) [1].

The remaining solid by-products can be classified as dust, sludge, and oily/non-oily mill scale. Traditionally, the majority of these by-products have been recycled back to the BF through the balling and sintering process [4,5,6,7]. However, 60% of the dust and sludge is very difficult to recycle without an extensive pretreatment due to the small particle size [8]. Sludge contains very fine hydrated dust from blast furnace dedusting and converter steelmaking [7,9,10,11]. The method of cold-bonded agglomeration is generally achieved by briquetting or pelletizing. Cold-bonded briquetting is superior to pelletizing when the raw material blend has a broad particle-size distribution with significant amounts of both fine and coarse particles [8]. There are methods of granulating or briquetting “fine dust” materials; for example, very fine dry particles (less than 0.1 µm) of material are granulated or briquetted after prior hydration and the addition of a binder. The granulation process is a continuous process, and the diameter of the pellets depends, in general, on the rotational speed and angle of inclination of the plate of the pelletizing machine and the efficiency of using a water spray as well as the speed and homogeneity of the dusty material. Pellets are solid, spherical bodies formed due to the interaction of iron ore concentrate particles and water. The magnitude of this effect depends on the properties of the material as well as its wettability and granularity. By selecting the speed of the pelletizing device and its angle of inclination, it is possible to obtain large pellets at the expense of a decrease in productivity, or the yield can be increased, but the diameter of the pellets is smaller.

Raw pellets are sometimes strengthened by roasting or cementation. Previous research [8,12] produced pellets with a diameter of 10 mm that were transported to an industrial plant on a tractor and used in the BOF process. The pellets were cold-bound using cement and contained about 12.5–13.5% moisture. Other researchers [13] studied iron ore fines from different mines to produce pellets for Corex and blast furnace plants. The iron ore fines were then ground in open-circuit ball mills to obtain the required fineness. To produce good-quality pellets, limestone was added in order to modify the pellets’ basicity. The strength of the produced pellets was studied, taking into account the different fineness of the dusts. In general, the strength of the pellets decreased as the size of the dust particles increased.

Fine metallurgical materials can be also agglomerated by briquetting in roller and stamp presses. Roller-briquetting machines are usually used in industry due to their advantages, the most important of which are significantly lower energy consumption, a longer life-cycle of formative components, and the continuous nature of the process, which makes it possible to achieve high efficiency.

In the case of slurries, the optimum moisture content and the type of binder material must be determined to produce pellets or briquettes of adequate strength. The authors of [14] took into account the effect of different binders on the strength of pellets. In [15], the authors tested molasses as a binder. In another paper [14], the authors examined starch and two components of adhesive–dry-hydrated-lime with molasses as a binder material. The briquetting process was carried out on an industrial production line equipped with a crusher, screen, conveyor belt, gravity storage tank, and roller-briquette machine. Briquettes with approximate dimensions of 50 × 20 mm were obtained. Their mechanical properties were determined from gravitational dump tests. The best briquettes were made from a mixture of waste with starch as a binder at a mass fraction of 3% in which the moisture content was approx. 8%. The only disadvantage of starch was the need for the long (about 100 days) seasoning of the material.

Another author [16] studied twenty-five combinations of dust with sludge and binders. The amount of dust varied between 0 and 80% in the samples, and the amount of sludge was in the range of 0–80%. The addition of the binders was between 10 and 40% of the total mass. The total volume of a briquette was 3.4 cm^3^, the roller velocity was 1.7 rpm, and the screw feeder rotational velocity depended on the force applied to the briquettes. The strength ranged from 10 kN to 100 kN. Finally, it was concluded that the physical and chemical compositions of the raw materials in the mixture and the pressure applied to the mixture in preparing the briquettes were important factors in attaining adequate resistance values for use in a blast furnace. The mechanical strength of the briquettes was also related to the effectiveness of the contact between the particles and binders, the fracture toughness of the phases present, and the homogeneous distribution of the binder in the mixture.

On the other hand, the authors of [17] analyzed nonlinear relationships between different factors that affect the briquetting process, including the content of plasticizer and moisture, hardness of the particles, dynamic viscosity of the liquid binder, amount of the carbonaceous component, and average particle size in the charge. It was shown that the hardness of the particles had the greatest influence on compressibility, while the dynamic viscosity of the liquid binder had the least effect.

Other researchers [18] have studied different types of briquetting machines suitable for the briquetting of fine-grained materials that are difficult to agglomerate, including iron-bearing materials.

From the point of view of the information mentioned above, the presented study researched the possibility of an efficient treatment of highly hydrated metallurgical sludge suspensions using an innovative microwave-based drying process.

Microwave-drying technology is considered to be fast, low-energy, and highly effective for the preprocessing of organic waste [19,20,21]. It should be noted that the microwave treatment of hazardous waste like sewage sludge or wastewater [22,23], medical waste [24], and even contaminated soil or radioactive graphite [25,26] has been widely studied in recent decades, but there is no mention in the scientific references of the use of microwave energy to dry metallurgical sludge.

In order to study the possibility of transporting sludge from landfills in the form of a hydrated suspension, the rheological properties of the mixes were examined.

This study approached the topic of recycling iron-bearing waste materials differently from those described in previous papers [8,9,10,11,12,13,14,15,16,17,18] by recycling not as briquettes or pellets, but as a suitably wet mixture for pouring into molds and then fast-drying in microwaves.

As fly ash (FA) from thermal power plants has pozzolanic properties, it was decided to use it as an additive in order to add mechanical strength to the cast material.

## 2. Materials and Methods

In total, 12 kg sludge from a landfill near the closed ArcelorMittal steel mill near Krakow and a sample of 4 kg fly ash from Krakow District Heating Company (DHC) (Kraków, Poland) were delivered. The materials were dried and four types of mixes were prepared according to the method shown in Table 1.

The addition of 10% fly ash as a hardener was selected based on a previous study [27], in which cement additions of 5% and 10% were tested. At that time, it was determined that 5% was sufficient, so—assuming that fly ash was weaker than cement—it was decided to double the added amount.

The rheological properties of the mixes were examined to study the possibility of transporting the sludge from landfills in the form of a hydrated suspension.

**Figure 1:**
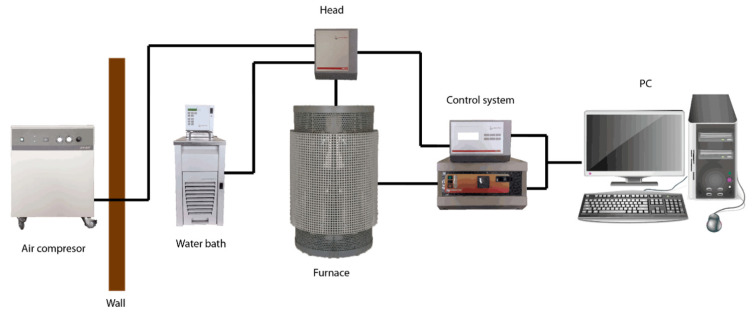
Schema of the high-temperature rheometer FRS1600 system.

**Figure 2:**
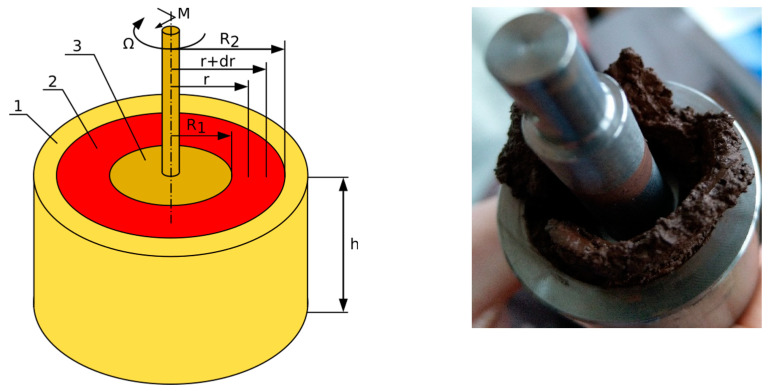
Concentric cylinder system. (**a**) Principle of rheometric study: 1—cup (outer cylinder); 2—measured sample; 3—bob (inner cylinder). (**b**) Concentrical cylinders after the experiment.

A rheological experiment was conducted using a high-temperature FRS1600 rheometer (Anton Paar GmbH, Graz, Austria) (Figure 1) with a standard head equipped with an air-bearing working in a concentric cylinder system using Searle’s method with a 7 mm gap (Figure 2).

The use of a rheometer with concentric cylinders, which used the principles and concepts of the Searle method, was primarily due to the versatility of this method. The obtained measurement results gave the most comprehensive rheological behavior characteristics of the tested systems.

After the calibration of the measurement system [28,29,30,31], the tests were conducted at room temperature.

The inner cylinder of radius R_1_ rotated at an angular speed of ω, the same as the layer of fluid adjacent to its surface. The outer cylinder had a radius of R_2_. When analyzing a system of coaxial cylinders, it is simplest to assume that the cylinders have infinite length, and only the part of the system with the height *h* is considered. When considering a fluid layer of a differential thickness *dr* located at distance *r* from the axis of rotation, the formula for the tangential force *Fr* in the fluid is given in Equation (1) [32].

(1) $$F_{r}=2\pi rh\tau_{r},$$

Here, *τ_r_* is the shear stress in the *r*-distance from the axis of the system.

Torque affected by the tangential force is ascertained by the formula given in Equation (2).

(2) $$M=F_{r}r,$$

It can be also expressed via Equation (3).

(3) $$M=2\pi hr^{2}\tau_{r},$$

After transforming Equation (3), a relationship can be obtained that allows the shear stress *τ_r_* at a distance *r* from the axis of rotation to be determined (Equation (4)).

(4) $$\tau_{r}=\frac{M}{2\pi hr^{2}},$$

This stress takes its maximum value on the surface of the inner cylinder when *r* = *R*_1_.

(5) $$\tau_{max}{=\tau }_{r}=\frac{M}{2\pi hR_{1}^{2}},$$

In turn, the minimum stress value is on the surface of the inner cylinder when *r* = *R*_2_.

(6) $$\tau_{min}{=\tau }_{r}=\frac{M}{2\pi hR_{2}^{2}},$$

The ratio of stresses from Equations (5) and (6) is equal to Equation (7).

(7) $$\frac{\tau_{max}}{\tau_{min}}=\frac{\tau_{1}}{\tau_{2}}=\frac{R_{2}^{2}}{R_{1}^{2}}=S^{2}$$

Here, *S* is the ratio of the radii of the measuring system.

The shear rate in the shear gap between the cylinders is related to the angular velocity, which varies with the distance from the *r*-axis (Equation (8)).

(8) $${\dot{\gamma }}_{r}=-\frac{rd\omega }{dr},\;$$

The shear rate value can be related to the shear stress values expressed by the formula in Equation (4).

(9) $$r={\left(\frac{M}{2\pi h\tau_{r}}\right)}^{\frac{1}{2}}={\left(\frac{M}{2\pi h}\right)}^{\frac{1}{2}}\;\tau_{r}^{-\frac{1}{2}},$$

After differentiating the relationship using Equation (8) concerning *τ_r_*, Equation (10) can be obtained.

(10) $$\frac{dr}{{d\tau }_{r}}={\left(\frac{M}{2\pi h}\right)}^{\frac{1}{2}}\left(-\frac{1}{2}\right)\tau_{r}^{-\frac{3}{2}},$$

After substituting the torque from Equation (3) into Equation (10), we obtain Equation (11).

(11) $$\frac{dr}{{d\tau }_{r}}={\left(\frac{2\pi hr^{2}\tau_{r}}{2\pi h}\right)}^{\frac{1}{2}}\left(-\frac{1}{2}\right)\tau_{r}^{-\frac{3}{2}}=\frac{-r}{2\tau_{r}},$$

After further transformations, we obtain Equation (12).

(12) $$\frac{dr}{r}=-\frac{d\tau_{r}}{2\tau_{r}},$$

The shear rate is a function of the shear stress and can be expressed as per Equation (13).

(13) $${\dot{\gamma }}_{r}=-r\frac{d\omega }{dr}=f(\tau_{r}),$$

After transforming Equation (13), Equation (14) is obtained.

(14) $$d\omega =-\frac{dr}{r}\;f\;(\tau_{r}),$$

After substituting this into Equation (12), Equation (15) is obtained.

(15) $$d\omega =\frac{1}{2}\;f\;(\tau_{r})\frac{d\tau_{r}}{\tau_{r}},$$

After integrating the relationship from Equation (15) within the limits for *r = R*_1_, *ω = Ω*, and *τ_r_ =τ*_1_, and for *r = R*_2_ and *τ_r_ = τ*_2_, Equation (16) is obtained.

(16) $$\int_{\omega =\Omega }^{\omega =0}d\omega =\frac{1}{2}\int_{\tau_{2}}^{\tau_{2}}f(\tau_{r})\frac{d\tau_{r}}{\tau_{r}},$$

The left side of the relationship can be directly integrated, which gives the following:

(17) $$\Omega =-\frac{1}{2}\int_{\tau_{2}}^{\tau_{2}}f(\tau_{r})\frac{d\tau_{r}}{\tau_{r}}$$

The above relationship is general.

The next stage of the research involved conducting experiments by drying samples Mix1–Mix4 in a microwave oven. A commercial household Samsung microwave oven with 1100 W max power was used. Each sample was cast into a silicon bakery mold with a maximum diameter of 5 cm and height of 2 cm; power from 100 to 600 W was applied during drying for 1–9 min.

To determine the mass of the tested samples, a RADWAG WTC 2000 (RADWAG Wagi Elektroniczne, Radom, Poland) precision balance was used, with a maximum load of 2000 g, a reading accuracy of 0.01 g, repeatability of 0.01 g, linearity of ± 0.03 g, a stabilization time of 2 s, and an operating temperature of 15–30 °C.

The cast sample was weighed and then microwaved for 1, 3, and 5 min; it was weighed again after each treatment to record the weight loss.

After determining the optimal drying program, lumps of approximately 20 mm in size were formed to test their compressive strength after drying in the microwave. Table 2 shows how the lumps were prepared. It should be noted that the amount of added water was selected so that the lumps could be manually formed; the main purpose of this operation was to determine the effect of an ash addition on the compressive strength of the formed material.

## 3. Results and Discussion

### 3.1. Material Characteristics

Table 3 presents the chemical analysis obtained from the samples of landfilled sludge and fly ash. It can be seen that the sludge contained a high iron content of about 63.7%. However, fly ash, thanks to a high gangue content ((SiO_2_ + Al_2_O_3_ + CaO) > 50%) and low carbon content (C < 16%) [33], was determined to have good pozzolanic properties and be as effective as cement in the lump-hardening process [27].

### 3.2. Rheological Experiment

Sludge is a suspension that is naturally characterized by a non-Newtonian flow. Moreover, it is a suspension that “precipitates” water under the influence of shearing; therefore, the study and analysis of the rheological behavior of a system using a wide range of shear rates is important, both from the point of view of the principal research and the development of technology to process waste material in the form of sludge into feedstock for extrusion, expression, and forming processes. The presented graphs illustrate the change in the flow of the sludge with different degrees of hydration and with different additives in order to fully characterize the tested substances.

The four prepared mixes were subjected to rheological measurements under changeable rheological conditions. The applied shear rate during rotational measurements was 5–50 s^−1^. The oscillation tests were carried out under conditions of a constant strain equal to 1% with a frequency of 1–100 Hz.

Firstly, the samples were stirred at a constant shear rate of 5 s^−1^ for 10 min. Then, the shear rate was increased to 10 s^−1^, again for 10 min. Finally, the measurements for the constantly changeable shear rate were obtained. These were from 5 to 50 s^−1^ over the first 150 s, then a decrease occurred from 50 to 5 s ^−1^ over the next 150 s.

**Figure 3:**
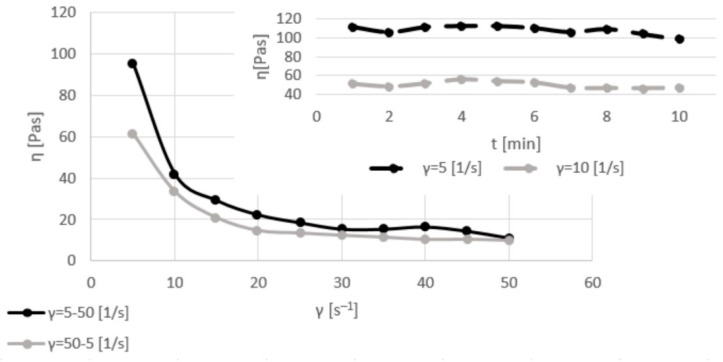
Viscosity curves of Mix1 for constant shear rates of 5 and 10 s^−1^ (dashed lines) and for various shear rates of 5–50 and 50–5 s^−1^ (solid lines).

**Figure 4:**
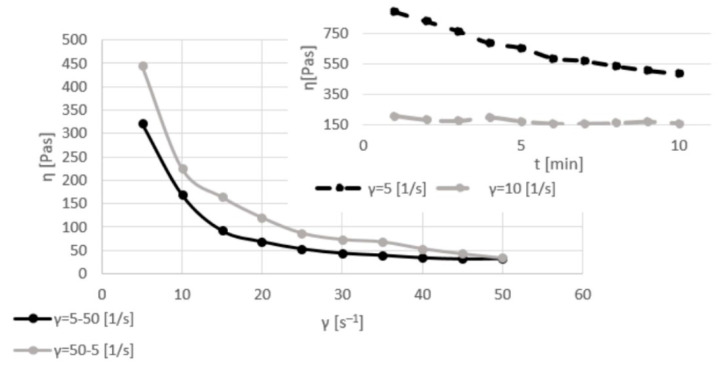
Viscosity curves of Mix2 for constant shear rates of 5 and 10 s^−1^ (dashed lines) and for various shear rates of 5–50 and 50–5 s^−1^ (solid lines).

**Figure 5:**
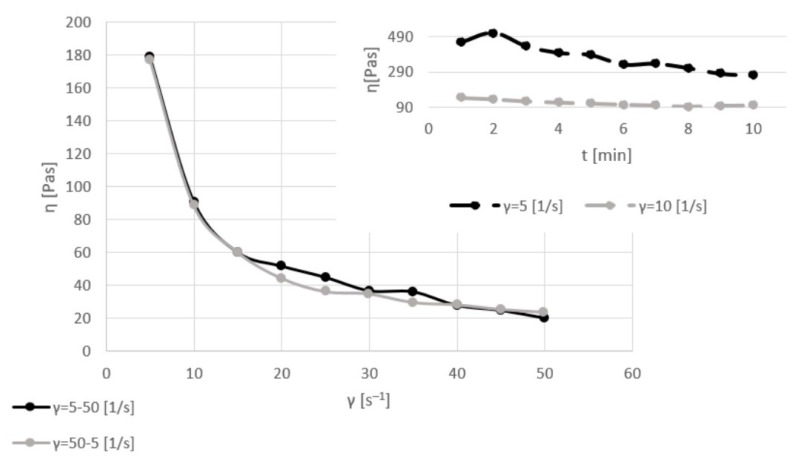
Viscosity curves of Mix3 for constant shear rates of 5 and 10 s^−1^ (dashed lines) and for various shear rates of 5–50 and 50–5 s^−1^ (solid lines).

**Figure 6:**
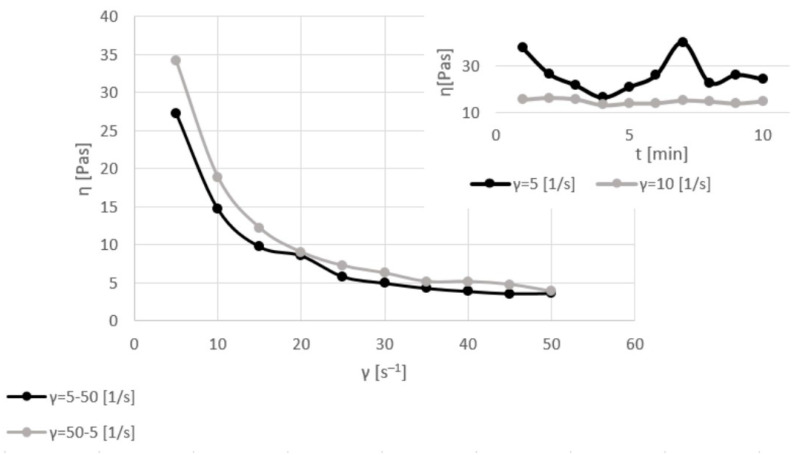
Viscosity curves of Mix4 for constant shear rates of 5 and 10 s^−1^ (dashed lines) and for various shear rates of 5–50 and 50–5 s^−1^ (solid lines).

Figure 3, Figure 4, Figure 5 and Figure 6 show the viscosity curves for the analyzed samples of Mix1–Mix4; the obtained viscosity data are summarized in Table 4.

Mix1’s highest dynamic viscosity coefficient values, about 110 Pas, were obtained while stirring with the lowest shear rate value, i.e., 5 s^−1^. The dynamic viscosity coefficient values slightly decreased during constant stirring with this force due to the effect of the homogenization of the sample. When applying a shear rate of 10 s^−1^, the system was characterized by lower and almost linear values of the dynamic viscosity coefficient, about 50 Pas. During shearing with a changeable force, the value of the dynamic viscosity coefficient decreased from ~100 to 10 Pas. Remixing from a high (50 s^−1^) to a low (5 s^−1^) shear rate showed that the inert structure of the system was rebuilt; however, the lower values of the dynamic viscosity coefficient for the same applied force were 100 Pas (for the “first cycle”) and 60 Pas for shearing at the rate of 5 s^−1^ (for the “second cycle”).

The samples containing both sludge and ash (Mix2) were characterized by higher values (850 Pas) of the dynamic viscosity coefficient than the raw sludge samples (110 Pas) with the same amount of water (31%) while stirring with the same shear rate (5 s^−1^). However, the behavior of the sludge–ash sample during mixing with a shear rate of 10 s^−1^ was similar to that of Mix1; the dynamic viscosity coefficient decreased (in comparison to the values for stirring at 5 s^−1^). During stirring using various shear rates (5–50 s^−1^), the value of the dynamic viscosity coefficient decreased from 320 to 40 Pas. Remixing from a high (50 s^−1^) to a low (5 s^−1^) shear rate showed that the inert structure of the system was rebuilt; some intermolecular interactions occurred, which resulted in an increase in the dynamic viscosity coefficient values for the same applied force of 450 vs. 40 Pas.

A rheological analysis of the behavior of a wetter (45%) sample of sludge with ash showed that the values of the dynamic viscosity coefficient were lower than those of the drier sludge–ash samples. The maximum value of the dynamic viscosity coefficient was about 490 Pas (for a shear rate of 5 s^−1^) and it decreased to 290 Pas while stirring for 10 min. The dynamic viscosity coefficient values for a shear rate of 10 s^−1^ were about 140–90 Pas. The viscosity curves for changeable shear rates of 5–50 and 50–5 s^−1^ almost overlapped; this indicated that the addition of 14% water changed the rheological behavior of the samples and that the values of the dynamic viscosity coefficient for the same shear rate were similar.

The raw sludge with the highest amount of water (60%; Mix4) showed the lowest value of the dynamic viscosity coefficient (38–24 Pas) for a shear rate of 5 s^−1^ and a moderately linear curve for a shear rate of 10 s^−1^ (viscosity of about 15 Pas). The result for the changeable shear rate also showed the lowest values of the dynamic viscosity coefficient of 3 vs. 27 Pas (for shear rates of 5–50 s^−1^) and 35 vs. 3 Pas (for shear rates of 50–5 s^−1^). The character of the curves for the changeable shear rate was similar to that of raw sludge with different amounts of water: lower values of the dynamic viscosity coefficient were always obtained during reverse mixing, which indicated that the inert structure of the samples had been rebuilt. However, the time was too short for a complete structure reconstruction.

Each sample was slowly homogenized at a constant shear rate of 5 s^−1^ for 10 min. In all cases (with different amounts of moisture, without and with the addition of ash), the value of the dynamic viscosity coefficient was highest at the beginning and slowly decreased during the first stirring. In the next step, the shear rate increased up to 10 s^−1^. When the samples were stirred twice as fast, the dynamic viscosity coefficient was much lower than at the beginning of the first mixing. Additionally, during shearing with higher values, the dynamic viscosity coefficient slightly changed and was almost linear during 10 min of stirring.

From a technological point of view, there are two options when operating with moist sludge. These are as follows:

1. Pre-mixing at a low shear rate between 5 and 10 s^−1^, then stirring with a rate of about 20 s^−1^ while pouring the sludge into molds.
2. Pre-mixing at a low shear rate between 5 and 10 s^−1^, stirring at a rate of about 50 s^−1^, and then pouring into molds.

However, considering the possibilities of transporting a sludge suspension by pumping, it should be noted that only Mix4 was suitable for this operation because it had the lowest dynamic viscosity coefficient.

### 3.3. Microwave Drying

**Figure 7:**
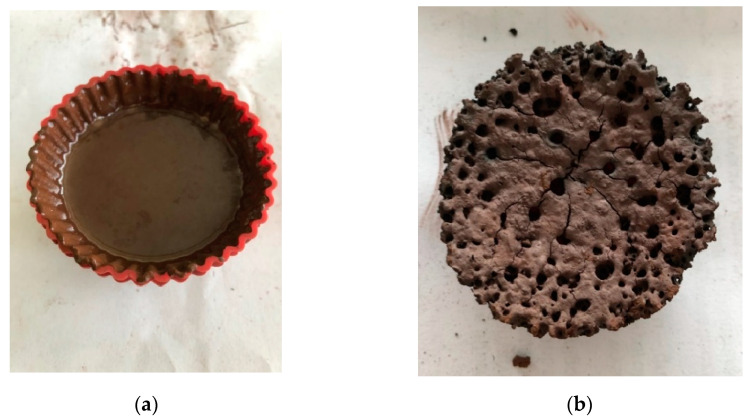
An example of a prepared and dried sample. (**a**) Freshly molded sample; (**b**) sample after microwave treatment.

Figure 7 presents a view of a wet and a dried sample.

Table 5, Table 6, Table 7 and Table 8 show the results of the microwave drying of the four examined mixes.

While drying Mix1, intense boiling was observed twice; when using a higher power, the weight loss exceeded the water content after 9 min (Table 5). This indicated the occurrence of chemical reactions such as the oxidation of carbon or the reduction of metal oxides. Thus, a further microwave treatment was omitted to prevent these processes at 600 W.

When drying Mix2, boiling was not observed, possibly due to the addition of fly ash and the resulting significant increase in the dynamic viscosity coefficient. It can also be observed from Table 6 that the weight of the samples took longer to decrease than Mix1.

Mix3 drying, however, was accompanied by three occurrences of intense boiling (Table 7). Moreover, the weight loss almost reached the water content in the sample (45%) in the last stage of treatment at a power value of 300 W. Thus, treatments at the power values of 450 and 600 W were shortened to 7 min.

When drying Mix4, boiling was observed in the middle treatment stage at a power value of 180 W; in the last stage, the sample exploded (Table 8). Therefore, treatment operations at a higher power were abandoned.

The following conclusions were drawn from the conducted studies: increased humidity and microwave power during drying could cause the samples in the molds to boil intensely, which, in extreme cases, could lead to them exploding. Apparently, rapid steam generation led to internal stress and the further destruction of the sample. Thus, further developments should consider using moderate drying powers (100–300 W), especially with high water contents (45–60%).

On the other hand, the addition of fly ash reduced the samples’ ability to boil.

### 3.4. Strength Tests

For the strength tests, it was decided to form the tested material into the shape of known input materials such as hematite pellets. The lumps were manually formed according to the methods shown in Table 2.

**Figure 8:**
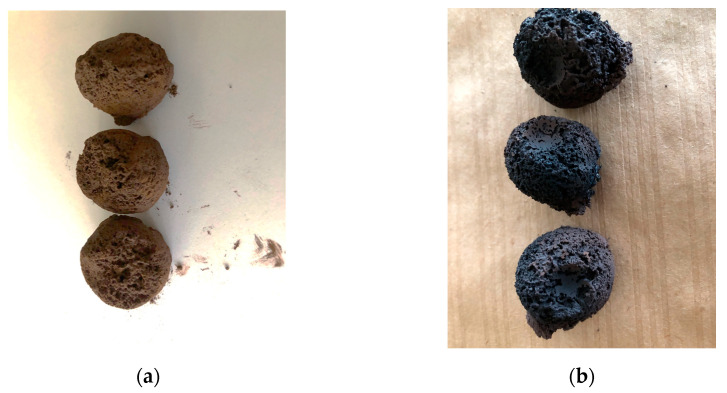
An example of manually formed and dried lumps. (**a**) Freshly made lumps; (**b**) lumps after drying at 300 W for 6 min in a microwave.

Drying experiments of these cast-into-mold samples showed that at an applied power of 300 W, a treatment time of 9 min was enough for complete drying. Thus, the lumps were dried at the same power, but the time of drying was shortened to 6 min because they contained one-third less water content than the cast-into-mold samples. Figure 8 depicts a view of the prepared lumps before and after the microwave treatment; Table 9 shows their compressive strength.

The obtained compression strength of the lumps was much lower than that of hematite pellets in the blast furnace process, the value of which is 20 N/psc in a green state and over 2000 N/psc after firing at 1100 °C. However, the intention of the present work was not to prepare a charge for a shaft furnace, but to ascertain the lumping and drying of sludge to make transportation possible to (preferably) nearby steel mills.

**Figure 9:**
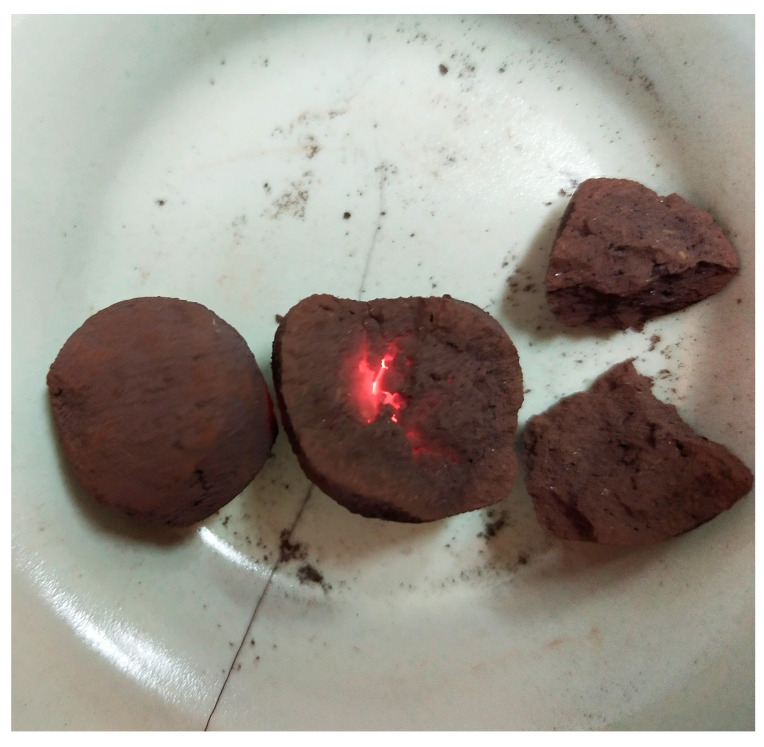
View of manually prepared wet lumps after microwave treatment at a power of 750 W.

Thus, wet lumps that were dried at 750 W for 2 min were treated to improve their strength value. Figure 9 presents the lumps after treatment.

Figure 9 shows that although the lumps glowed inside from the high temperature, they probably cracked because the water evaporated too quickly. Therefore, in the next experiment, the lumps were dried at a power of 300 W for 6 min; then, without taking them out of the oven, they were treated at 750 W for 1 min.

It can be seen (Table 10) that the additional treatment enhanced the compression strength by about 30% for Lump1 and 95% for Lump2, which indicated the favorable use of fly ash as a strengthening additive. This was consistent with other authors’ [34] conclusions that several types of zeolites are rapidly formed during fly ash microwave treatments, thus strengthening the internal structure and increasing the lump compression strength.

**Figure 10:**
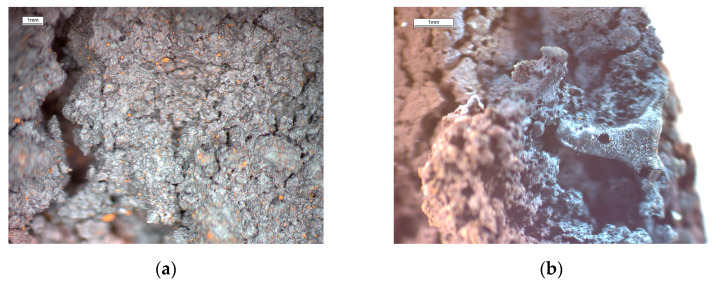
Stereoscopic microscope photos of cores: (**a**) Lump1 (sludge only); (**b**) Lump2 (sludge with fly ash).

Figure 10 depicts photos of the cores of Lump1 and Lump2 taken with an Opta-Tech stereoscopic microscope (Opta-Tech sp. z o.o., Warsaw, Poland). It can be observed that the core was sintered or even melted due to a microwave treatment with a higher power.

## 4. Conclusions

The main goal of the present study was the development of new technology to prepare sludge suspension materials using a microwave-drying process in order to produce a charge for a further metallurgical reduction.

This study demonstrated that microwave drying at 300 W for 6 min was optimal for sludge processing, achieving a weight loss of 35% and total drying. A further higher-power microwave treatment at 750 W cause compressive strength improvements up to 15.83 N/psc with the addition of fly ash.

Future work could focus on exploring alternative binders and scaling up the process for industrial applications.

Based on the experiments and tests, the following stages of sludge processing technology are proposed:

1. At a landfill site, the sludge should be hydrated up to 60% of water content in order to obtain a consistency of suspension that can be pumped to the processing factory.
2. Preliminary drying is needed in the factory. This can be performed by sedimentation and water squeezing using filter presses until the water content drops to about 30–40%.
3. Next, up to 10% of another dry waste (i.e., fly ash) could be added to the sludge, after which the sludge mixture can be cast into molds.
4. Microwave-heating at medium power for 6–9 min should be used; then, if necessary, at the highest power for 1–2 min.

These operations should allow dry material to be obtained that can withstand transportation to steel mills, preferably nearby, where they could be further processed metallurgically.

## Figures and Tables

**Table 1:** Mass proportion of studied mixes.

	Mix1	Mix2	Mix3	Mix4
Landfilled sludge, %	69	59	45	40
DHC fly ash, %	–	10	10	–
Water, %	31	31	45	60

**Table 2:** Mass proportion of prepared lumps.

	Lump1	Lump2
Landfilled sludge, %	79	69
DHC fly ash, %	–	10
Water, %	21	21

**Table 3:** Chemical analysis of mixture components.

	SiO_2_	Al_2_O_3_	Fe_2_O_3_	Fe_3_O_4_	CaO	MgO	SO_3_	Alkalis	Zn	C	H_2_O	LOI
Landfilled sludge	4.3	1.5	11.7	71.4	5.4	0.8	–	0.5	2.5	1.7	29.4	4.1
DHC fly ash	48.9	20.7	12.6	–	5.8	2.5	1.0	2.7	–	5.6	–	9.8

**Table 4:** Maximum and minimum values of the dynamic viscosity coefficients of the studied mixes for various shear rate conditions.

Shear Rate γ, s^−1^	Max/Min Value of Dynamic Viscosity Coefficient η, Pas			
	Mix1 (FA = 0%, H_2_O = 31%)	Mix2 (FA = 10%, H_2_O = 31%)	Mix3 (FA = 10%, H_2_O = 45%)	Mix4 (FA = 0%, H_2_O = 60%)
5	115/100	850/480	490/290	38/24
10	60/50	170/150	140/90	16/14
5–50	100/10	320/40	180/20	27/3
50–5	60/10	450/40	180/23	35/3

**Table 5:** Change in masses using different microwave-oven powers and drying times for sample Mix1.

Microwave-Oven Power, W	Initial Mass, g	Weight Loss, %		
		1 min	4 min	9 min
100	76.08	0.10	1.25	7.66
180	76.35	0.15	7.98 *	23.69
300	73.54	1.75	16.90 *	32.94
450	75.87	4.03	30.43	35.40
600	73.91	8.89	30.82	–

* Intense boiling was observed.

**Table 6:** Change in masses using different microwave-oven powers and drying times for sample Mix2.

Microwave-Oven Power, W	Initial Mass, g	Weight Loss, %		
		1 min	4 min	9 min
100	75.52	0.03	0.79	6.68
180	75.91	0.13	6.96	22.29
300	74.36	1.19	14.74	28.50
450	75.41	2.48	25.16	–
600	74.13	5.42	28.23	–

**Table 7:** Change in masses using different microwave-oven powers and drying times for sample Mix3.

Microwave-Oven Power, W	Initial Mass, g	Weight Loss, %			
		1 min	4 min	7 min	9 min
100	73.81	0.09	0.58	–	4.65
180	74.95	0.09	4.43	–	22.53 *
300	75.05	0.31	17.09 *	–	41.10
450	75.11	1.07	32.01 *	46.76	–
600	74.19	3.52	39.76 *	46.01	–

* Intense boiling was observed.

**Table 8:** Change in masses using different microwave-oven powers and drying times for sample Mix4.

Microwave-Oven Power, W	Initial Mass, g	Weight Loss, %		
		1 min	4 min	9 min
100	49.63	0.06	0.67	5.59
180	49.27	0.10	29.65 *	**

* Intense boiling was observed. ** The sample exploded.

**Table 9:** Weight loss and compression strength of lumps dried in microwaves.

Parameter	Lump1	Lump2
Average initial mass of lump, g/psc	11.2	9.9
Weight loss after drying, %	20.87	17.54
Average compression strength, N/psc	9.42	8.10

**Table 10:** Compression strength of lumps after complex microwave treatment.

Parameter	Lump1	Lump2
Average compression strength, N/psc	12.37	15.83

## Data Availability

The original contributions presented in the study are included in the article, further inquiries can be directed to the corresponding author.
